# Effects of Explant Source and Orientation on Secondary Somatic Embryogenesis in *Hevea brasiliensis*

**DOI:** 10.3390/plants14213274

**Published:** 2025-10-27

**Authors:** Xiaochuan Gu, Jingyu Ao, Lisheng Kong, Xuemei Dai, Huasun Huang, Huabo Du, Xiaoyi Wang, Tiandai Huang

**Affiliations:** 1 Key Laboratory of Biology and Genetic Resources of Rubber Tree, Ministry of Agriculture and Rural Affairs, National Key Laboratory for Tropical Crop Breeding, Rubber Research Institute, Chinese Academy of Tropical Agricultural Sciences, Haikou 571101, China; guxiang28@163.com (X.G.); xuemeidai@126.com (X.D.); huanghuasun@catas.cn (H.H.); 2 Haikou Key Laboratory of Tropical Plant Seedling Innovation, Haikou 571101, China; 3 College of Tropical Crops, Yunnan Agricultural University, Puer 665099, China; a2734001929@163.com (J.A.); duhuabo1976@163.com (H.D.); 4 Centre for Forest Biology, Department of Biology, University of Victoria, Victoria, BC V8W 3N5, Canada; lkong@uvic.ca

**Keywords:** embryogenic capacity, histological study, rubber tree, starch, superoxide dismutase activity, soluble protein, soluble sugar

## Abstract

Propagation of rubber tree (*Hevea brasiliensis*) via secondary somatic embryogenesis (SSEis) is a reliable method. However, its efficiency is relatively low. The aim of this study was to understand more about the factors related to SSEis in rubber trees, trying to improve the efficiency of somatic embryo (SE) yield. Our study showed that the orientations of explants, i.e., the fragments of primary SE (PSE), on the medium affected secondary SE (SSE) yield significantly. Among five experimental tests, the highest yield was 2.6 ± 0.9 secondary somatic embryos (SSEs) per explant, which was achieved by orienting the abaxial side of the explant in contact with the medium and then the adaxial side after a period of culture time. Based on histological evidence, SSEis was induced from the epidermal cells and adjacent cells on the adaxial side of the explants. A remarkable difference in embryogenic capacity difference existed among individual PSE. The concentrations of soluble proteins, starch, soluble sugars, and the superoxide dismutase activity (SOD) levels in the explants were measured during a 25-day long SSEis induction treatment and compared between explants of high and low embryogenic capacity. This study proves that the explant orientation toward the culture medium plays a crucial role in SSEis, while the concentration changes of these biochemical compounds correlate to morphological changes in the explants during induction, as do the changes in SOD activity. Furthermore, the trend of the dynamic changes in the explants reflected a process of de-differentiation and re-differentiation, which started from mature SE tissues during SSE induction.

## 1. Introduction

The rubber tree (*Hevea brasiliensis*) is a perennial cross-pollinated species belonging to the genus *Euphorbiaceae*. The natural rubber produced by rubber trees has the characteristics of high elasticity, insulation, air tightness, eco-friendliness, and resistance to wearing, oil, and extreme temperatures. The products of natural rubber are widely used in industry, agriculture, national defense, transportation, and daily life. Since natural rubber, as an important raw material, is mainly produced by rubber trees, there is an urgent need for research and technological advancements to propagate elite rubber trees.

Somatic embryogenesis (SEis) is the process by which somatic cells develop into embryos without the fusion of gametes. These embryos, or somatic embryos (SEs), can convert into plants under appropriate conditions. This is regarded as the most efficient method to propagate elite woody plants [1,2]. Besides mass propagation, SEis is also an efficient system for genetic engineering, breeding, and conservation of higher plants [1]. Chinese and Malaysian groups initiated the study of SEis research in rubber trees in the 1970s [3]. The first rubber tree SEs were obtained by Paranjothy in 1974, using the anther wall as explants [4]. SEs obtained in this way were successfully converted into plantlets and established in soil a few years later by Wang et al. in 1977 [5].

In plants, mature SEs could be produced through either indirect or direct SEis. The former is regarded as more efficient, by which embryogenic callus or tissue is induced first and the embryos were then generated from this kind of tissue. However, the plants produced with this method showed noticeable abnormalities in field-planted rubber plants [6,7], which possibly resulted from genetic variations and epigenetic changes after many repeated callus subcultures [8]. Thus, the intermediate callus phase should be restricted, in order to minimize the risk of somaclonal variations [6]. In 2010, Hua et al. [9] developed a reliable method for producing rubber trees via SSEis. First, primary SEis (PSEis) was induced using the anthers of rubber tree as explants. Then, the mature primary SEs (PSEs) were cut into small pieces, or embryonic fragments, and used as explants to induce SSEis. In this case, SSEs developed directly from this kind of explant. The multiplication rate was 13, i.e., 13 SSEs were produced from one PSE [9]. This process can be carried on for a few cycles or whenever needed. Plant regeneration was finally achieved with mature SSEs. The conversion rate from SSEs into plants reached 86% or higher [9,10]. Most importantly, the rubber trees produced with this method showed performance in the growth and rubber yield that was similar to the mother trees. In addition, few genetic differences were found in trees obtained via SSEis, when compared with the mother trees [11,12,13]. As a result, rubber tree somatic embryo-derived plantlets were produced mainly by using the SSEis method in Hainan, China [10]. Theoretically, an unlimited quantity of mature embryos can be produced via the repeated cycles of SSEis. However, the production efficiency of SSEs was relatively low in addition to genotype limitations. These factors resulted in high production costs and restricted the large-scale propagation of rubber trees via SSEis in practice [14]. At present, there are few reports on system optimization for rubber tree propagation via SSEis.

Previous reports indicated that in the SSEis system of rubber trees, a majority (>50%) of somatic embryos exhibited developmental arrest during the early morphogenetic stages (globular, heart, and torpedo), and were unable to successfully develop into cotyledonary embryos [13]. As in other higher plants, the explant source is one of the key factors for successfully inducing SEis or SSEis in rubber trees [15]. Besides the genetic sources of the explant, it was reported that significant differences existed in SE initiation frequency from either the abaxial or adaxial surface when the cotyledons or leaves were used as explants [16,17]. Also, it was noticed that the embryogenic capacity of each PSE could be remarkably different when used as the explant to induce SSEis. In previous reports, some biochemical parameters were regarded important to SEis in higher plants, such as the levels of sugars, starch, proteins, superoxide dismutase activity, etc. in the plant tissues [18,19,20].

This study investigated the effects of culture method and explant source on SSEis in rubber trees, including optimization of culture conditions and histological and biochemical analyses of changes in explants during a 25-day long SSEis induction treatment. Based on the research results, the SSE production system was improved remarkably, with a higher SSE multiplication rate that was fourfold higher than the rate reported previously by Hua et al. [9].

## 2. Results

### 2.1. Primary and Secondary Somatic Embryogenesis

**Figure 1:**
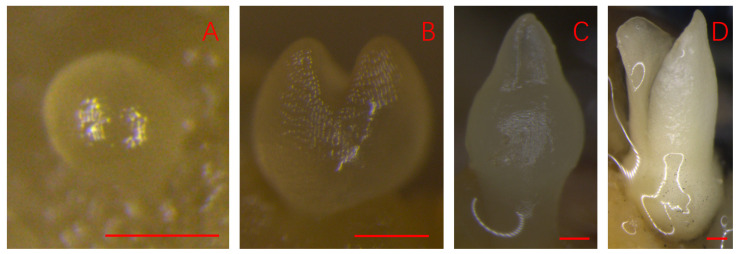
Four developmental stages of PSE in rubber trees. (**A**) A globular embryo on embryogenic callus; (**B**) a heart-shaped embryo; (**C**) a torpedo-shaped embryo; (**D**) a cotyledonary embryo. Bars = 0.2 mm.

**Figure 2:**
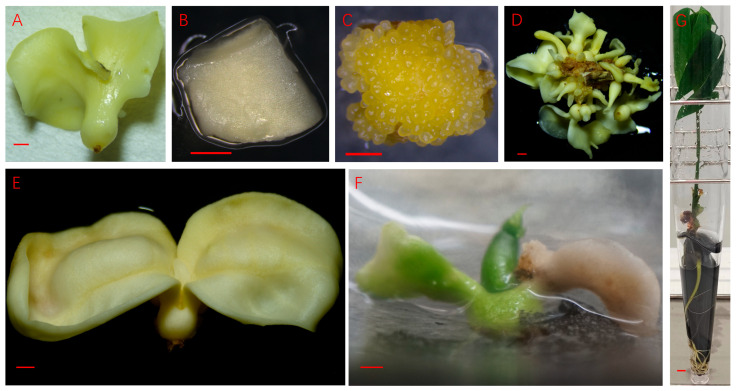
The major steps for inducing secondary somatic embryogenesis in rubber trees. (**A**) A mature primary somatic embryo (the explant source); (**B**) a piece of cotyledon cutting (the explant); (**C**) an explant with numerous globular embryos on the surface 26 days after culture initiation; (**D**) cotyledonary secondary somatic embryos generated from an explant cultured for 49 days on embryo development medium; (**E**) an isolated secondary somatic embryo; (**F**) a germinating secondary somatic embryo; (**G**) a secondary somatic embryo plantlet. All bars = 1 mm. Scale bars are shown in each photo. Scale bars (**A**–**F**) 1 mm; (**G**) 0.5 mm.

The PSEis was induced using the anthers as explants. During the induction, the embryogenic callus, which is usually hard in texture, occurred first and the primary embryos of spherical shape developed on the embryogenic callus. Explants with the induced embryogenic callus and tiny globular embryos on the surface (Figure 1A) were then transferred to the embryo development medium to stimulate embryo development and maturation (Figure 1B–D). The cotyledons of mature PSEs (Figure 2A) were excised and cut into small pieces (Figure 2B), which were used as explants to induce SSEis. SSEs were induced directly from the explants without a callus phase (Figure 2C). Similar to PSE, the induced embryos passed through four developmental stages. i.e., spherical shaped or globular embryos, heart-shaped embryos, torpedo-shaped embryos, and cotyledonary embryos (Figure 1A–D) before the embryos reached full morphological maturity (Figure 2D). Isolated SSEs were transferred to a germination medium in test tubes, where they germinated and developed into plantlets (Figure 2E–G).

### 2.2. Effects of Explant Orientation on SSEis

The influence of explant orientation on SSEis was investigated using five experimental groups of cotyledonary explants (Table 1). Groups 1 and 4 involved switching the adaxial/abaxial side contacting the medium upon transfer to the embryo development medium. In contrast, Groups 2 and 3 maintained the original orientation. Group 5 explants were oriented randomly. All visible SSEs across every experimental group were collectively counted as “Total SSE” after a 45-day development culture, irrespective of their size. The induction rate of SSE in Group 1 explants was the highest among all groups, and was significantly higher (*p* < 0.05) than that in Group 2, Groups 4 and 5 (Table 1). As shown in the table, the best result was obtained from Group 1 of the experiments, in which the explant was cultured with its abaxial side in contact with the medium first and then the adaxial side. The induction rate was 2.6 ± 0.9 SSEs per explant, which was twofold higher when compared to those of Groups 3 and 5, i.e., the explant side in contact with the medium was consistently the adaxial side, or it was placed randomly on the medium. It was 4 or 5-fold higher than the induction rate of Group 1 when compared with those of Groups 2 or 4, respectively. In all five explant groups, the SSEs were exclusively induced from the adaxial side.

### 2.3. Morphological and Histological Changes During SSE Induction Process

**Figure 3:**
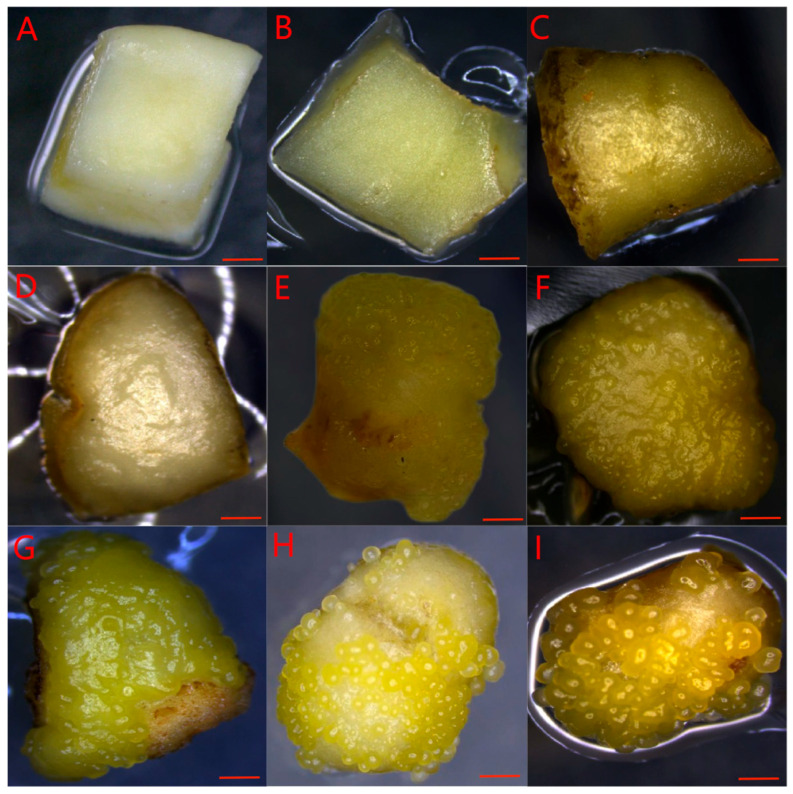
The morphology of rubber tree explants on culture medium for induction of secondary somatic embryogenesis. (**A**–**I**) The explants were cultured on induction medium at day 0, 3, 6, 9, 12, 15, 18, 21, or 24, respectively. (**A**) The surface of the explant was smooth and milky white in color. (**B**,**C**) The explant changed into light yellow and became swollen within 6 days of culture. (**D**,**E**) The explant surface then became uneven with tiny bumps. (**F**–**I**) Numerous globular SSE appeared on the surface two weeks after the beginning of induction treatment. All scale bars = 0.5 mm.

Morphological and histological analysis was carried out in Group 1 explants, which were cultured with their abaxial side in contact with induction medium. When the induction treatment started at day 0, the surface of the explant was smooth and milky white in color (Figure 3A). The explant changed into light yellow and became swollen within 6 days of culture (Figure 3B,C). The explant surface then became uneven with tiny bumps (Figure 3D,E). Numerous globular SSEs appeared on the adaxial surface two weeks after the beginning of induction treatment (Figure 3F–I).

**Figure 4:**
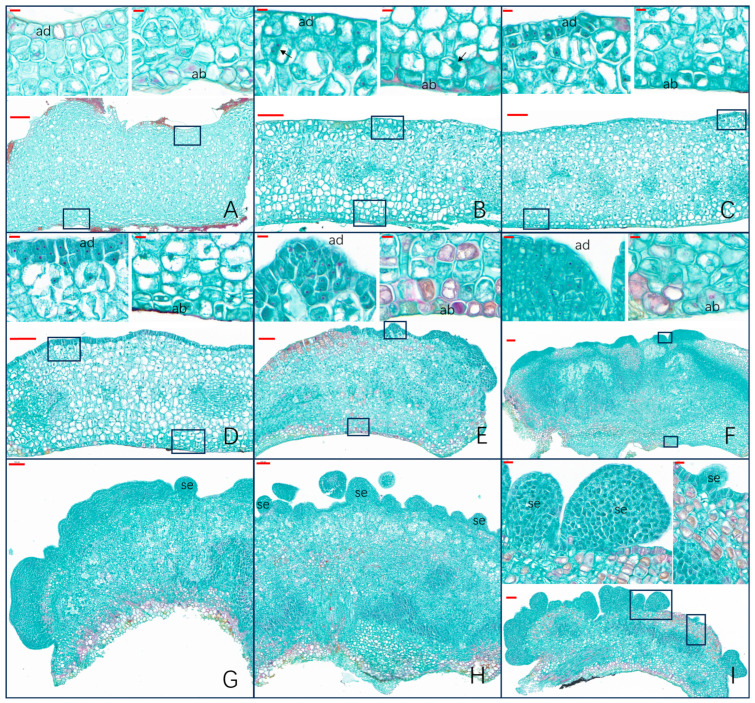
Histological changes in Group 1 rubber tree explants on culture medium for induction of secondary somatic embryogenesis. (**A**–**I**) The explants were cultured on induction medium at day 0, 3, 6, 9, 12, 15, 18, 21, or 24, respectively, and the upper side is the adaxial side in all the graphs. (**A**,**B**) On days 0 and 3, the epidermal layers of adaxial and abaxial sides appeared similar, with only sporadic cell divisions (arrows). (**C**,**D**) On days 6 and 9, the adaxial epidermal cells (with a high nuclear–cytoplasmic ratio, dense cytoplasm, and deep staining) divided periclinally and anticlinally, whereas only a few dividing abaxial cells were observed. (**E**) On day 12, cell division on the adaxial side formed multiple layers, whereas the abaxial side showed limited development, with cells becoming necrotic. (**F**,**I**) Globular embryos and embryonic cell clusters in the adaxial surface increased in size and number. Abbreviations: (ad) adaxial side, (ab) abaxial side, (se) somatic embryo. Scale bars are shown in each photo. Scale bars (**A**–**I**) overall micrographs 100 μm; (**A**–**F**) magnification micrographs 10 μm; (**I**) magnification micrographs 20 μm.

Under a light microscope, the cross section of the explant showed no obvious difference in the epidermal layers between the adaxial and abaxial side on day 0 and day 3. Only a few cell divisions occurred (Figure 4A,B). On day 6 and day 9, the epidermal cells on both the adaxial and abaxial side, along with the adjacent subepidermal cells, exhibited periclinal and anticlinal divisions (Figure 4C,D). The dividing adaxial epidermal cells, which were small, tightly arranged, deeply stained, and had a high nuclear–cytoplasmic ratio, primarily underwent periclinal divisions in some areas. In contrast, there were fewer dividing cells in the abaxial epidermal layer (Figure 4D). At the adaxial side, the epidermal cells and adjacent subepidermal cells kept dividing, resulting in multiple cell layers. Cells in these layers were tightly arranged and exhibited meristematic features, such as dense cytoplasm and large nuclei (Figure 4E,F). On the other hand, the abaxial side showed limited development after day 12, with cells becoming necrotic (Figure 4E). By that time (day 12), embryonic cell clusters appeared in the epidermis of the adaxial surface of the explant. Thereafter, these structures and globular embryos increased in size and number (Figure 4F–I).

### 2.4. Biochemical Analysis of the Explants During SSE Induction

#### 2.4.1. Soluble Protein Levels in the Explants of High or Low Embryogenic Capacity

**Figure 5:**
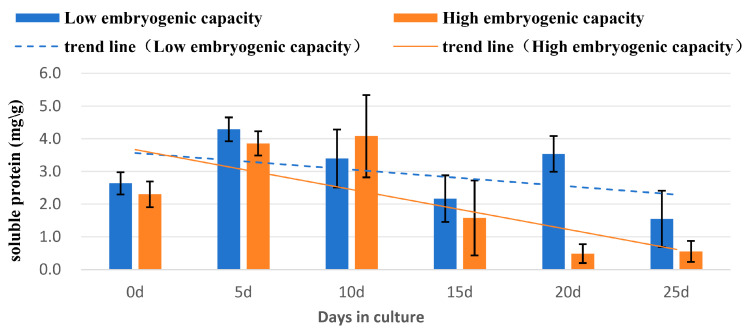
Changes of soluble protein concentration in the explants of high and low embryogenic capacity during induction of SSEis, mean ± SD, *n* = 3.

Concentrations of soluble proteins increased after the explants were placed onto the induction medium, and the highest concentrations were reached at day 5 or day 10 in the samples of high or low embryogenic capacity, respectively (Figure 5; Table 2). The concentrations then declined to the lowest values at day 20 or day 25 in the samples of high or low embryogenic capacity, respectively. The decline was two times higher in the sample of low embryogenic capacity and eight times higher in the sample of high embryogenic capacity compared with their respective highest concentrations. At day 20, a significant difference (*p* < 0.05) in the concentrations was found between the samples. At this time point, the level in the low embryogenic capacity sample was seven-fold higher than that of the high embryogenic capacity (Table 2).

#### 2.4.2. Starch Concentrations

**Figure 6:**
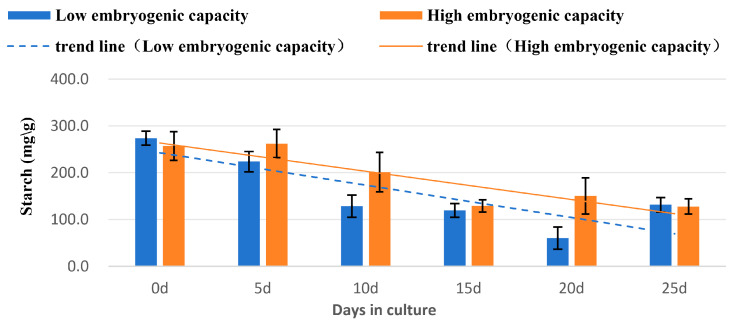
Changes of starch concentration in the explants of high and low embryogenic capacity during induction of SSEis, Mean ± SD, *n* = 3.

The highest levels of starch existed at day 0 and day 5 in the samples of high embryogenic capacity, and at day 0 in the low embryogenic capacity. Starch levels declined as a general trend in both kinds of samples (Figure 6). Significantly higher starch concentrations (*p* < 0.05) were detected in the high embryogenic capacity sample than those in the low concentration on both days 10 and 20, whereas the concentrations were similar in both of the samples at day 25 (Table 3).

#### 2.4.3. The Changes of Soluble Sugar Concentration

**Figure 7:**
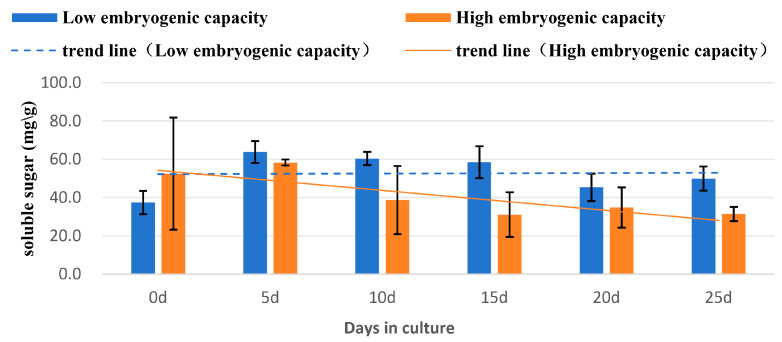
Changes of soluble sugar concentration in the explants of high and low embryogenic capacity during induction of SSEis, mean ± SD, *n* = 3.

The concentration of soluble sugar was higher in the sample of high embryogenic capacity than that in the sample of low embryogenic capacity at day 0. The levels increased sharply to the highest at day 5 in the samples of low embryogenic capacity, then decreased slowly from day 10 to day 25. In the high embryogenic capacity samples, levels increased slightly to a peak at day 5; thereafter, they decreased significantly by day 15 and remained at this level until day 25. The decline was deeper with the samples of high embryogenic capacity than those of low embryogenic capacity, which was a decrease of 1.9 or 1.3 times at day 25, respectively (Figure 7). Also, significantly lower concentrations (*p* < 0.05) were found at day 15 in the sample of high embryogenic capacity compared to the low sample (Table 4).

#### 2.4.4. Activity of Superoxide Dismutase (SOD)

**Figure 8:**
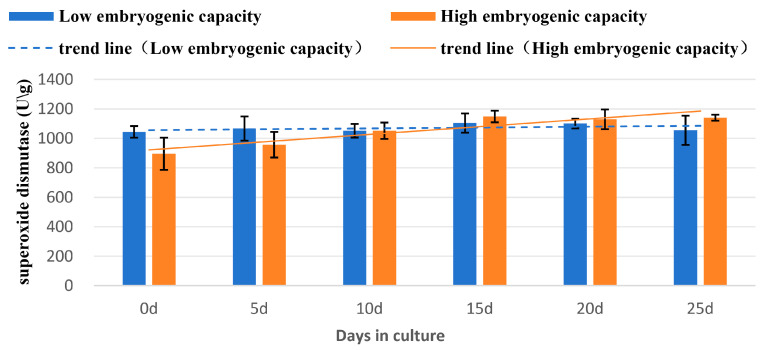
Changes of superoxide dismutase activity in the explants of high and low embryogenic capacity during induction of SSEis, mean ± SD, *n* = 3.

The SOD levels were significantly lower in the sample of high embryogenic capacity than those in the sample of low embryogenic capacity at day 0, and then increased significantly in the sample of high embryogenic capacity by day 10 and remained at this level until day 25, while there was no significant change in the low embryogenic capacity. During the entire induction treatment, the general trend of SOD level in the samples of low embryogenic capacity declined slightly, or was unchanged. However, an obvious increase occurred in the samples of high embryogenic capacity (Figure 8; Table 5).

## 3. Discussion

### 3.1. Effects of Explant Orientations on SSE Yield

This study shows clearly that the explant orientation on the culture medium plays an important role in inducing SSEis in rubber trees. With the best culture method, i.e., the abaxial side toward to the culture medium first and then the adaxial side to the medium, the SSE yield per explant increased to 2.6 SSEs, or 4-fold SSE yield when compared with the previously reported, which was 0.6 SSEs per explant [9]. It was reported that the orientation of the explants affected somatic embryogenesis in many species [21,22,23]. The gradient of plant growth regulators (PGRs)/phytohormones between the two sides of the explant may favorite SSE initiation when the abaxial side contacted the medium first, whereas when the adaxial side toward the medium, the developing SSEs could absorb nutrients directly from the culture medium, which better supports embryo growth. Without the PGR gradient in rubber tree explant tissue, the SSEs could not initiate, which was supported by the fact that SSEis could not be induced if the explants were submerged in the induction liquid culture medium in our other studies. During the somatic embryo development stage of this study, when the abaxial side of the explants was placed in contact with the medium (as in Group 2 and Group 4), only the embryos on the periphery of the explants in direct contact with the medium developed into the cotyledonary stage. In contrast, non-peripheral embryos fail to further develop due to the absence of direct contact with the embryo development medium. This finding accounts for the previously reported issue that over half of the proembryos ceased development prior to the cotyledon stage [13]. Based on the data presented in Table 1, it is evident that a higher number of mature SSEs were generated in Group 1. Although the difference between Group 1 and Group 3 was not statistically significant, the method used in Group 1 remains the most efficient in practical applications. By adopting this approach, we have successfully resolved the long-standing challenge of achieving sustainable propagation of SSEs in other rubber tree clones, including Reyan 917, Reyan 879, Reken 628, and PR107.

The preference of SEis started from one side of the explant, usually the adaxial side, was reported in many species [16,17,24], which could result from the difference between the two sides of the explant in structural and/or physiological conditions. Our results demonstrate that SSE induction was uniformly restricted to the adaxial side across all five explant groups, irrespective of which side contacted the medium. This is consistent with observations from our earlier studies, in which the occurrence of SSEs on the abaxial side was a very rare event.

### 3.2. Origin of Secondary Somatic Embryos on Explants

In this study, direct SSEs were induced from adaxial side of the explant, which is similar to the process in *Phalaenopsis* [25]. This spatial specificity suggests that the adaxial and abaxial sides of the cotyledons possess distinct capacities or follow divergent pathways for embryogenesis. Supporting this notion, in Arabidopsis, somatic embryos develop on the adaxial side, while the abaxial side typically forms a callus [26]. Although previous research in rubber trees indicated that SSEs mainly originate from epidermal cells [13], our findings further refine this by demonstrating that this potential is restricted solely to the adaxial epidermis.

Previous reports have described various pathways for SEis, including single-cell origin [27,28,29], multicellular origin [30,31], or both within the same species [17,32]. In rubber trees, both unicellular and multicellular origins of SEis have been documented [13,33,34], with the specific pathway influenced by genotype–medium interactions and subject to experimental control [35]. Although our histological analysis captured early stages of SSE initiation during induction treatment, we could not definitively determine whether SSEs arose from single cells or cell groups. However, based on the embryo–explant interface, most SSEs appeared to originate from multiple cells, with only a minority likely deriving from a single cell. This observation is further supported by a recent gene-editing study in rubber trees using the SSEis system, in which 90% of edited embryos were chimeric [36], strongly indicating a predominantly multicellular origin. Elucidating the precise cellular origins of SE will require the integration of multiple approaches—such as single-cell or thin-layer culture systems, combined with biochemical and molecular analyses.

### 3.3. Changes of Biochemical Levels

Our study revealed the trend of level changes of sugar, starch, soluble protein, and SOD in the explants during induction of SSEis in rubber trees. The increase of soluble sugar from day 0 to day 5 was possibly due to sugar being absorbed by the explants from the sugar-rich culture medium. However, the increase of soluble protein levels from d0 to d5 may result from biosynthesis for these needs. In both the levels of soluble sugar and soluble protein, the declines occurred from d5 and lasted until d25. The trends of decline were sharper in the explant of high embryogenic capacity than the low embryogenic capacity (Figure 5 and Figure 7). Soluble proteins and other storage compounds like soluble sugars and starch are essential for cellular metabolism and stress responses during SEis [37,38]. These compounds can provide the necessary energy and structure materials for cell growth, division, and differentiation during SEis. In coffee (*Coffea arabica* L.), the initial explant for inducing SEis was leaves. The trends of several biochemical components, such as soluble sugars and proteins, were increasing in the developing SEs [20]. Unlike the trend of increasing levels in these biochemical compounds during SE initiation and development [20,39], the trend revealed in this study was decreasing, which reflects a process of de-differentiation of the explants, which were embryonic instead of vegetative pieces, and re-differentiation for SSE initiation. The physiological changes were more active in the explant of high embryogenic capacity.

Starch is one of the storage substances that commonly exist in embryonic cells [40]. Its level increases during SE development [18,20]. In this study, the higher level of starch was found in the tissue of high embryogenic capacity, although the levels of starch declined in the explant during the SE induction process.

Superoxide dismutase is an antioxidant enzyme that is common in aerobic organisms. It functions in defending against damage to the cell membrane system by free radicals [19] and quickly accumulates during embryo development to protect tissues and cells [41]. In this study, during SSEis induction treatment, the SOD level kept increasing, which was positively correlated with the induction of SSEs. The peaks were 1149 U/g and 1104 U/g at d15, respectively, in the samples of high and embryogenic capacity, when the globular embryos started to form. The increasing SOD level in the samples of high embryogenic capacity indicates that the higher levels of SOD favorite SSE initiation.

## 4. Materials and Methods

### 4.1. Plant Materials

Immature anthers at the uninucleate stage were collected from the unopened male flowers of rubber tree clone Reyan 73,397 at the experimental field of the Chinese Academy of Tropical Agricultural Sciences (Danzhou, Hainan, China) and used as explants. The unopened male flowers are yellow-green in color and have a longitudinal diameter of approximately 3 mm.

### 4.2. Producing Primary Somatic Embryos

Somatic embryogenesis was induced from immature anthers. Primary somatic embryos were induced, developed and matured following the steps described in the previous report [14]. The mature primary somatic embryos were further used as explants for inducing secondary somatic embryogenesis.

### 4.3. Inducing Secondary Somatic Embryogenesis

The entire mature PSEs, excluding the radicle end, were cut into small pieces with an area of approximately 4–9 mm^2^ (2–3 mm × 2–3 mm). On average, a total of 30 pieces could be obtained from each PSE and used as explants for the induction. Explants (10) were placed into each Petri plate containing the culture medium. The abaxial side of the explants was placed in contact with the culture medium for the induction [42]. Cultures were kept under darkness, 22–24 °C for 26–30d before data collection. The induction medium for embryogenesis was made using modified MS medium salts plus 1.5 mg/L kinetin, 1.5 mg/L 2,4-D, 1.5 mg/L naphthalene acetic acid, 0.1 g/L inositol, 0.1 g/L hydrolyzed casein, 0.3 g/L asparagine, 70 g/L sucrose, 50 mL/L coconut water, and 2.2 g/L gelling gum (Phytagel, Sigma-Aldrich, St. Louis, MO, USA) [9]. The medium pH was adjusted to 5.8 before autoclave. In this study, all the organic medium components, plant hormones and growth regulators were purchased from Shanghai Bioengineering Technology Co., Ltd., Shanghai, China and/or Sigma-Aldrich, St. Louis, MO, USA [14].

### 4.4. Secondary Somatic Embryo Development

After 26–30d induction culture, the explants that generated embryonic callus or globular embryos were selected under a dissection microscope, transferred to the embryo development medium, and placed on the medium with its adaxial side in contact with the medium to stimulate embryo development. Cultures were kept under darkness, 24–26 °C for 42–49d before data collection. The embryo development medium contained the modified MS salts and 1.0 mg/L BAP, 3.0 mg/L kinetin, 0.5 mg/L gibberellic acid, 0.06 mg/L 2,4-D, 0.1 g/L inositol, 70 g/L sucrose, 50 mL/L coconut water, 2.2 g/L gelling gum, and 1 g/L activated charcoal [9]. Medium pH was adjusted to 5.8 before autoclave.

### 4.5. Study on the Effects of Explant Orientations

In this experiment, cotyledon-derived explants were positioned on the induction medium in specific orientations to investigate the effect of explant orientation on induction rate. In Group 1, all the explants were initially cultured with their abaxial side in contact with the medium. After 26 days, the explants were reoriented to position the adaxial side in contact with the embryo development medium. In group 2, all the explants were initially cultured with their abaxial side in contact with the medium. After 26 days, the explants were kept the same orientation to position the abaxial side in contact with the embryo development medium. In Group 3, all the explants were initially cultured with the adaxial side in contact with the medium. After 26 days, the explants were kept the same orientation to position the adaxial side in contact with the embryo development medium. In Group 4, all the explants were initially cultured with their adaxial side in contact with the medium. After 26 days, the explants were reoriented to position the abaxial side in contact with the embryo development medium. In Group 5, the explants were initially randomly cultured with their adaxial side or abaxial side in contact with the medium. After 26 days, the explants were randomly oriented to position the adaxial side or abaxial side in contact with the embryo development medium. The medium components and the culture conditions were the same as described in Section 4.3 and Section 4.4. Data was collected after the completion of the secondary somatic embryo development culture. The experiment was repeated three times. The visible cotyledonary SSEs of total explants for each experiment, regardless of size, were counted together as total SSE. SSE/Expt of each experiment is equal to total SSE divided by the total explants of each experiment. The mean ± SD of each group is equal to the mean and standard deviation of the three repeated experimental SSE/Expt.

### 4.6. Histological Study on Secondary Embryogenesis

Mature primary somatic embryos of high embryogenic capacity were used as the explant source. Their cotyledons were cut into embryonic blocks and used as explants to induce SSEis. The process and culture conditions were the same as described in Section 4.3. Samples for histological study were collected once every 3 days, i.e., at day 0, 3, 6, 9, 12, 15, 18, 21, and 24. The samples were fixed immediately with the FAA fixative (50% ethanol) and stored in a refrigerator at 4 °C for future use. For sectioning, the preserved samples were dehydrated in an ethanol series (60, 70, 80, 90, and 100%), before being infiltrated with paraffin wax and then embedded in paraffin blocks. The block with sample was then sectioned into slices of 8–10 μm in thickness with a rotary microtome. Paraffin was removed before the slices were stained with Safranin O-Fast Green (Wuhan Servicebio Technology Co., Ltd.). The slides were then covered with cover slips and examined under a light microscope (Leica, Wetzlar, Germany). The selected slices were photographed using a Leica CCD DMF 500 camera (Leica, Wetzlar, Germany).

### 4.7. Methods for Physiological and Biochemical Analysis

#### 4.7.1. Sample Collection According to Embryogenic Capacity

Briefly, each mature PSE was numbered and cut into small pieces (2–3 mm × 2–3 mm). All the pieces from each embryo were placed separately on the induction medium in a numbered Petri plate. In this experiment, a total of 30 embryos were used to cut into pieces and were used as explants. Samples were collected from each plate at different time points, i.e., 0, 5, 10, 15, 20, and 25 days of the induction culture. At each time point, the samples from each explant embryo were quickly frozen with liquid nitrogen and then stored individually in an ultra-low temperature refrigerator at −80 °C before analysis.

The number of explants that generated globular embryos in the remaining explants of each PSE were counted using a microscope starting 15 days after the culture was initiated. The induction rate of explants with globular embryos of each PSE was calculated separately with the following formula. If the induction rate of explants was 50–90%, these PSE were regarded as of high embryogenic capacity, whereas the PSE of low embryogenic capacity responded poorly: only 0–30% of the total explants could generate globular embryos. The previously frozen samples were then grouped for analysis according to the standard for high and low embryogenic capacity.

Induction rate of explants (%) = number of explants with globular embryos/total number of remaining explants of each PSE × 100%

#### 4.7.2. Analysis of Soluble Protein

Soluble protein in the samples was measured with Coomassie Bright Blue Method, using a total protein quantitative test box (Nanjing Jiancheng Bioengineering Institute Co., Ltd., Nanjing, China). The sample, approximately 20 mg FW, was homogenized in a buffer with an ice water bath for protein extraction. The absorbance value was recorded at a wavelength of 595 nm using a spectrophotometer (Persee TU-1810, Persee Co. Ltd., Beijing, China). A standard curve was created with standard protein of a series of known concentrations. The protein concentration in the sample was calculated by using the standard curve and the following formula based on the absorbance reading. Analysis for each treatment was carried out three times with different samples as replicates.

Protein concentration in the sample (mg/g) = (OD test − OD blank)/(OD standard−OD blank) × standard protein concentration × dilution factor

OD: optical density

#### 4.7.3. Starch Analysis

A starch detection kit (micro method, Beijing Solebao Technology Co., Ltd., Beijing, China) was used to check starch content. Starch was extracted from the sample (30 mg FW) and measured. Three samples from each treatment were used as replicates. Readings were performed in a spectrophotometer at 620 nm. Starch concentrations were determined using a range of glucose concentrations as standards. Starch content in the sample was calculated using the following formula.

Starch concentration in the sample (mg/g) = (A × V − extraction/W)/1.11 × F

V-extraction: volume after extraction, mL; W: sample weight, g; F: fold of sample dilution; 1.11: the conversion constant of the glucose content into the starch content.

#### 4.7.4. Analysis of Soluble Sugar

Soluble sugar content was measured by using a plant soluble sugar content test box (colorimetric method, Nanjing Jiancheng Bioengineering Institute Co., Ltd., Nanjing, China). Soluble sugar was extract from a sample of 20 mg FW. Readings were performed in a spectrophotometer at a wavelength of 620 nm. The value was recorded and calculated for soluble sugar content in the sample by comparison with soluble sugar standards using the following formula. Three samples from each treatment were measured as replicates.

Soluble sugar content in the sample (mg/g FW) = (OD test − OD blank)/(OD standard − OD blank) × C-standard)/(W/V mention) × N

C-standard C: The concentration of soluble sugar standard; W: the fresh weight of the tissue, g; V: the total volume of the added extract (distilled water), mL; N: the dilution factor of the sample before the test.

#### 4.7.5. Determination of Superoxide Dismutase (SOD) Activity

Superoxide dismutase (SOD) activity was measured by using a plant SOD kit (hydroxyl method, Nanjing Jiancheng Bioengineering Institute Co., Ltd., Nanjing, China). The measurement was made three times with three samples from each treatment, and each sample included plant tissue of 20 mg FW. The absorbance value was recorded at a wavelength of 450 nm, and the superoxide dismutase (SOD) activity in the sample was calculated with the following formula.

SOD vitality (U/G tissue) = SOD inhibition rate/50% × reaction system (0.24 mL)/dilution factor (0.02 mL) × N/(G/M)

SOD inhibition rate = [(OD control−OD control blank) − (OD measurement − OD measurement blank)]/(OD control − OD control blank) × 50%

N: Sample dilution factor; G: the sample weight, g; M: the added homogenizing buffer, mL.

### 4.8. Data Analysis

Excel 2020 was used for organizing data; WPS Office 2022 for graphing and mapping; SPSS 23.0 for single-factor analysis of variance and multiple comparisons (Data analysis of the explant orientation’s effects on SSE using Tukey’s HSD; biochemical data analysis of the explants during SSE induction using Duncan test).

## 5. Conclusions

Somatic embryogenesis could be an efficient technique for the propagation of rubber trees, which offers the advantages over other methods, such as clonal uniformity, rapid multiplication, genetic improvement, etc. However, SSEis in rubber trees is a complex process and requires the optimization of various factors, including explant selection and culture method optimization. This study reports a more efficient method for SSE production via SSEis in rubber trees. The method of placing the explants, or the fragments of PSE, in contact with the culture medium significantly affected SSE yield. The best treatment was to position the abaxial side of the explant in contact with the medium and then the adaxial side after a period of culture time. Histological study showed that the SSEis started from epidermal cells at the adaxial side of the explants. Investigations of the changes in biochemical levels reveal a process that may differ from other SE systems. Since the explants were pieces of PSEs, this kind of tissue went through a de-differentiation course during the induction treatment before generating globular SEs. Some differences were found between the explants of high or low embryogenic capacity. The changes in the levels of soluble sugar, soluble protein, and SOD were more obvious in the high embryogenic capacity tissue than the low one.

## Figures and Tables

**Table 1:** Effects of culture methods on the yields of SSEs.

Group of Experiments (Explant Side Contacting Medium)		Total Explants	Total SSE	SSE/Expt	Mean ± SD
Group 1 (Abaxial side→Adaxial side)	Exp 1-1	75	129	1.7	2.6 ± 0.9 (a)
	Exp 1-2	75	267	3.6	
	Exp 1-3	72	189	2.6	
Group 2 (Abaxial side→Abaxial side)	Exp 2-1	77	32	0.4	0.7 ± 0.2 (b)
	Exp 2-2	72	59	0.8	
	Exp 2-3	85	60	0.7	
Group 3 (Adaxial side→Adaxial side)	Exp 3-1	78	50	0.6	1.4 ± 0.7 (ab)
	Exp 3-2	74	153	2.1	
	Exp 3-3	83	115	1.4	
Group 4 (Adaxial side→Abaxial side)	Exp 4-1	75	35	0.5	0.6 ± 0.1 (b)
	Exp 4-2	75	48	0.6	
	Exp 4-3	84	49	0.6	
Group 5 (Randomly→Randomly)	Exp 5-1	84	61	0.7	1.1 ± 0.4 (b)
	Exp 5-2	84	131	1.6	
	Exp 5-3	84	86	1.0	

Different letters in the table indicate differences based on statistic analysis (*p* < 0.05). SD stands for standard deviation.

**Table 2:** Changes of soluble protein content in explants of high and low embryogenic capacity during induction of SSEis *.

Days of Culture	Soluble Protein Content (mg/g FW)					
	0	5	10	15	20	25
Embryogenic capacity (Low)	2.6 ± 0.3	4.3 ± 0.4	3.4 ± 0.9	2.2 ± 0.7	3.5 ± 0.6	1.6 ± 0.9
	(bcde)	(a)	(abcd)	(de)	(abc)	(ef)
Embryogenic capacity (High)	2.3 ± 0.4	3.9 ± 0.4	4.1 ± 1.3	1.6 ± 1.1	0.5 ± 0.3	0.6 ± 0.3
	(cde)	(ab)	(a)	(ef)	(f)	(f)

* Values represent mean ± SD, *n* = 3. Different letters in the table indicate statistically significant differences (*p* < 0.05).

**Table 3:** Changes of starch concentration in explants of high and low embryogenic capacity during induction of SSEis *.

Days of Culture	Starch Concentration (mg/g FW)					
	0	5	10	15	20	25
Embryogenic capacity (Low)	273.7 ± 15	223.7 ± 22	128.2 ± 24	119.3 ± 15	60.1 ± 24	131.6 ± 15
	(a)	(bc)	(d)	(d)	(e)	(d)
Embryogenic capacity (High)	256.9 ± 31	262.3 ± 30	201.2 ± 42	129.3 ± 13	150.1 ± 39	127.6 ± 16
	(ab)	(ab)	(c)	(d)	(d)	(d)

* Values represent mean ± SD, *n* = 3. Different letters in the table indicate statistically significant differences (*p* < 0.05).

**Table 4:** Changes of soluble sugar content in explants of high and low embryogenic capacity during induction of SSEis *.

Days of Culture	Soluble Sugar Content (mg/g FW)					
	0	5	10	15	20	25
Embryogenic capacity (Low)	37.4 ± 6.0	63.8 ± 5.7	60.4 ± 3.5	58.4 ± 8.3	45.3 ± 7.2	49.9 ± 6.3
	(cd)	(a)	(ab)	(abc)	(abcd)	(abcd)
Embryogenic capacity (High)	52.4 ± 29.3	58.3 ± 1.6	38.7 ± 17.8	31.1 ± 11.6	34.8 ± 10.5	31.4 ± 3.7
	(abcd)	(abc)	(bcd)	(d)	(d)	(d)

* Values represent mean ± SD, *n* = 3. Different letters in the table indicate statistically significant differences (*p* < 0.05).

**Table 5:** Changes of superoxide dismutase activity in explants of high and low embryogenic capacity during induction of SSEis *.

Days of Culture	Superoxide Dismutase Activity (u/g FW)					
	0	5	10	15	20	25
Embryogenic capacity (Low)	1044 ± 39	1067 ± 83	1052 ± 46	1104 ± 65	1101 ± 32	1055 ± 99
	(ab)	(ab)	(ab)	(a)	(a)	(ab)
Embryogenic capacity (High)	895 ± 108	956 ± 87	1052 ± 55	1149 ± 40	1130 ± 67	1140 ± 20
	(c)	(bc)	(ab)	(a)	(a)	(a)

* Values represent mean ± SD, *n* = 3. Different letters in the table indicate statistically significant differences (*p* < 0.05).

## Data Availability

The original contributions presented in this study are included in the article. [insert reason here].

## Abbreviations

The following abbreviations are used in this manuscript:

SEis	Somatic embryogenesis
SE	Somatic embryo
SEs	Somatic embryos
PSEis	Primary somatic embryogenesis
PSE	Primary somatic embryo
PSEs	Primary somatic embryos
SSEis	Secondary somatic embryogenesis
SSE	Secondary somatic embryo
SSEs	Secondary somatic embryos
SOD	Superoxide dismutase activity
OD	Optical density
